# Glycyrrhizic Acid Scavenges Reactive Carbonyl Species and Attenuates Glycation-Induced Multiple Protein Modification: An *In Vitro* and *In Silico* Study

**DOI:** 10.1155/2021/7086951

**Published:** 2021-10-11

**Authors:** Sahir Sultan Alvi, Rabia Nabi, Mohd. Shahnawaz Khan, Firoz Akhter, Saheem Ahmad, M. Salman Khan

**Affiliations:** ^1^IIRC-5, Clinical Biochemistry and Natural Product Research Lab, Department of Biosciences, Integral University, Lucknow, 226026 U.P., India; ^2^Department of Biochemistry, College of Sciences, King Saud University, Riyadh, Saudi Arabia; ^3^Department of Biomedical Engineering, Stony Brook University, New York, USA; ^4^Department of Medical Laboratory Sciences, College of Applied Medical Sciences, University of Hail, Hail City, Saudi Arabia

## Abstract

The current study is aimed at studying the inhibitory effect of glycyrrhizic acid (GA) on D-ribose-mediated protein glycation via various physicochemical analyses and in silico approaches. Being a potent free radical scavenger and a triterpenoid saponin, GA plays a vital role in diminishing the oxidative stress and thus could be an effective inhibitor of the nonenzymatic glycation process. Our data showed that varying concentrations of GA inhibited the *in vitro* BSA-AGEs via inhibiting the formation of fructosamines, fluorescent AGEs, scavenging protein carbonyl and hydroxymethyl furfural (HMF) content, and protection against D-ribose-induced modification of BSA as evident by increased free Arg and Lys residues in GA-treated Gly-BSA samples. Moreover, GA also attenuated D-ribose-induced alterations in the secondary structure of BSA by protecting the *α*-helix and *β*-sheet conformers and amide-I band delocalization. In addition, GA attenuated the modification in *β*-cross amyloid structures of BSA and in silico molecular interaction study too showed strong binding of GA with higher number of Lys and Arg residues of BSA and binding energy (*Δ*G) of -8.8 Kcal/mol, when compared either to reference standard aminoguanidine (AG)-BSA complex (*Δ*G: -4.3 Kcal/mol) or D-ribose-BSA complex (*Δ*G: -5.2 Kcal/mol). Therefore, GA could be a new and favorable inhibitor of the nonenzymatic glycation process that ameliorates AGEs-related complications *via* attenuating the AGE formation and glycation-induced multiple protein modifications with a reduced risk of adverse effects on protein structure and functionality; hence, it could be investigated at further preclinical settings for the treatment and management of diabetes and age-associated complications.

## 1. Introduction

Diabetes is a group of metabolic disorders characterized by long-term hyperglycemia which in turn leads to the initiation and progression of severe diabetes-associated ailments [1–3]. The nonenzymatic glycosylation, a multistep reaction, implicates excessive chemical attachment of sugars to proteins, nucleic acids, and lipids without the involvement of enzymes, resulting in the generation of early glycation products (EGPs), known as Amadori products, which on further oxidation generates dicarbonyl compounds to form irreversible and highly reactive cross-linked structures known as advanced glycation end products (AGEs) [4, 5]. The production of AGEs remains an irreversible phenomenon and continues to accumulate indefinitely in various body tissues that results in further cross-linking or modifying intracellular and extracellular proteins and in generating reactive oxygen radicals [6]. Thus, the exorbitant formation and accumulation of AGEs in the tissues is a significant contributor to diabetic ailments such as nephropathy, retinopathy, and neuropathy [1, 7, 8].

Various strategies have been considered to inhibit the glycation reaction due to the complex processes leading to AGE formation involving several pathways, intermediates, and end products. However, the amino groups (mostly lysine or arginine) make proteins more susceptible to glycation-induced oxidative modification resulting in structural alterations and functional changes [9, 10]. Many proteins like albumin from bovine serum (BSA) and human serum (HSA), as well as low-density lipoprotein (LDL), were previously analyzed for their glycation-induced oxidative alterations [4, 5, 8]. Albumin is directly exposed to the alterations in glucose level which make them a very susceptible molecule for glycation. It has been identified that glycation induces modification of 34 lysine residues (out of 59) of albumin as well as amyloid-like fibrillation of albumin which further exhibits impaired functions [5, 11]. On the other hand, from our previous studies, it is clearly validated that upon *in vitro* glycation of albumin, AGEs are produced more quickly with D-ribose [4, 5, 8]. D-ribose, a reducing monosaccharide, is a major constituent of many key biomolecules and produces glycated adducts (AGEs) when it reacts with albumin [4, 8].

Through various scientific studies, it has been well documented that inhibition of nonenzymatic glycosylation reaction can be a beneficial approach to counter secondary complications of chronic diabetes. Many pharmacological approaches, together with the use of inhibitors, were established to constrain the glycation reaction and consequent glycation signaling pathway [4, 5, 12, 13]. Several agents, natural or chemically synthesized, which have been suggested as glycation inhibitors are either limited to the *in vitro* studies or are related with unwanted complications [5, 12, 13]. Considering the side effects of synthetic compounds, there is a need for an alternative approach to prevent nonenzymatic glycation [4, 5, 12].

**Figure 1:**
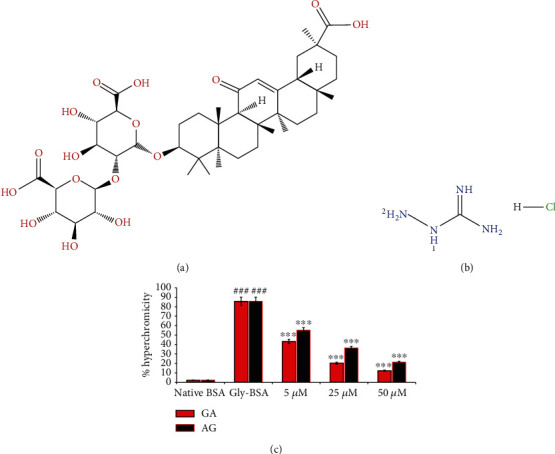
(a) 2D structures of glycyrrhizic acid (PubChem ID: 14982). (b) 2D structures of aminoguanidine hydrochloride (PubChem ID: 14982). Retrieved from PubChem database (https://pubchem.ncbi.nlm.nih.gov/). (c) % hyperchromicity in native BSA, Gly-BSA, and GA-/AG-treated Gly-BSA. The data represented the mean ± SEM of three independent experiments. Significant difference vs. native BSA at ^###^*p* < 0.001 and vs. Gly-BSA at ^∗∗∗^*p* < 0.001.

Nonenzymatic glycation reaction can be delayed in the human system by adopting robust lifestyles that includes minimum exposure to oxidants, radiation, smoke, and oxidized foods as well as consumption of antioxidant-rich diet [1, 4]. Natural products having numerous pharmacological activities were evaluated *in vitro* and *in vivo* for their capability to prevent distinct ailments along with the AGE formation [3–5, 14–17]. One of the biologically active compounds is glycyrrhizic acid (GA), which is getting attention nowadays and is a major constituent found in the root of the genus Glycyrrhiza (Leguminosae) or licorice plants [18]. The chemical structures of GA (a triterpenoid saponin) and aminoguanidine (AG) are illustrated in Figures 1(a) and 1(b), respectively. GA is well known for its exceptional pharmacological properties including antioxidant, stimulant, anti-inflammatory, antigastric ulcer, antihepatotoxic, and antiviral properties [18–21]. There are few reports that mentioned the antidiabetic activity of GA in animals [22], and only one report depicted the beneficial effect of GA on the AGE/RAGE axis in an animal model [23]. As yet, no scientific research elaborates the role of GA in attenuating nonenzymatic glycation-induced structural alterations of protein by using various biochemical/biophysical and in silico approaches. Thus, based on the above descriptions, the current work was meant to decipher the preventive property of GA against the EGPs and AGEs as well as the beneficial effect with respect to glycation-mediated multiple protein modifications, particularly amyloid fibrillation and protein aggregation by implying various physicochemical and in silico molecular interaction studies.

## 2. Materials and Methods

8-Aninilo-1-napthalene sulphonate (ANS), D-ribose, thiobarbituric acid (TBA), congo red (CR), sodium dihydrogen phosphate, 2,4-dinitrophenyl-hydrazine (DNPH), BSA, nitro-blue tetrazolium (NBT), sodium chloride, guanidine hydrochloride, and disodium hydrogen phosphate were procured from Hi-Media labs, India. 2,4,6-Trinitrobenzene-1-sulphonic acid was obtained from G-Biosciences. Standard drug AG, 9,10-phenanthrenequinone, thioflavin-T (ThT), and GA were purchased from Sigma Aldrich Co. USA.

### 2.1. In Vitro BSA Glycation/Antiglycation Assay

The *in vitro* glycation of BSA was performed using D-ribose under sterile condition. In short, BSA (0.5 mg/mL) was incubated with D-ribose (80 mM) in 100 mM phosphate buffer saline (PBS) (pH 7.4) at 37°C, containing 0.05% sodium azide with and without addition of GA and AG (reference antiglycating agent) at a concentration of 5, 25, and 50 *μ*M, respectively, for 18 days. The sample mixture was then dialyzed thoroughly against PBS to eliminate all unrelated constituents. The reactive mixture with BSA and 80 mM D-ribose (Gly-BSA) was used as a glycation control, and native BSA was used as a control. The doses of BSA, D-ribose, and AG were chosen based on formerly established protocols [4, 5]. However, the GA concentration used in this study was chosen based on previously published report showing that GA is nontoxic and safe up to 1000 *μ*M [24, 25]. The GA and AG were dissolved in ddH_2_O.

### 2.2. Physicochemical Analysis and Characterization

#### 2.2.1. Investigation of Hyperchromicity via UV-Vis Spectroscopy

It is well known that protein glycation results in increased absorbance due to various glycation adduct formation, referred to as hyperchromicity [4, 5]. Hence, we analyzed the significant changes in BSA's absorption pattern on glycation or antiglycation for 18 days regularly using the Eppendorf BioSpectrometer. The spectral analysis of native and Gly-BSA with and without AG/GA (5, 25, and 50 *μ*M) was studied using a wavelength of 200-800 nm. The belowmentioned equation was used to determine the glycation inhibitory activity of GA and AG, and results were obtained in terms of hyperchromicity percentage at 280 nm [4, 5]: %hyperchromocity = [(Absorbance of glycated sample − Absorbance of native or Inhibitor‐treated sample)/Absorbance of glycated sample] × 100.

#### 2.2.2. Confirmation of Amadori Products via NBT Reduction Assay

Protein glycosylation results in the Amadori product formation or ketoamines that initiate further rearrangement and cyclisation reactions leading to the AGE formation. These ketoamines are known as early glycation products (EGPs). Thus, the NBT assay was performed daily (for 18 days) to determine EGP generation in native and Gly-BSA with and without addition of GA and AG at 5, 25, and 50 *μ*M concentrations using Eppendorf BioSpectrometer [5, 26]. In brief, 20 *μ*L of native, Gly-BSA, GA, and AG (5, 25, and 50 *μ*M) samples were added to 180 *μ*L NBT (0.25 mM) dye dissolved in sodium carbonate-bicarbonate buffer (100 mM, pH 10.8) and incubated for 10 min at 37°C, and the OD was measured at 525 nm.

#### 2.2.3. Analysis of Carbonyl Content

In all the samples, carbonyl content (CC) was assessed by DNPH assay as previously described [4, 5]. The absorption was recorded at 360 nm, and the molar extinction coefficient of 22,000 M^−1^ cm^−1^ was used to interpret the data as DNPH reacted/mg protein [5].

#### 2.2.4. Determination of HMF Content via TBA Assay

The hydroxymethylfurfural (HMF) content of all the samples mentioned above were determined by TBA assay. In each of the (100 *μ*L) samples mentioned above, 400 *μ*L oxalic acid was mixed, boiled for 1 h, and cooled for 10 min. After that, the reaction mix was precipitated by putting TCA, 500 *μ*L (40%), and kept for centrifugation at 8000 rpm (24°C) for 10 min. Following the centrifugation, supernatant was collected, and 500 *μ*L of TBA (0.05 M) was mixed and incubated for 1/2 h at 40°C. The absorption was measured at 443 nm using TBA as blank. The HMF content was calculated using a molar extinction coefficient of 40,000 M^−1^ cm^−1^ [4, 5].

#### 2.2.5. Detection of Intrinsic Fluorescent AGEs by Fluorescence Spectroscopy

Fluorescence spectra was recorded by exciting the samples, i.e., native and Gly-BSA, in the presence or absence (5, 25, and 50 *μ*M) of GA and AG at 370 nm on fluorescence spectrophotometer. The fluorescence emission intensity (FI) was recorded at 360-600 nm, and the percentage variation in FI for all of the samples was calculated using the equation below [27]: %Increase/Decrease in FI = [(FI of glycated sample − FI of native or inhibitor‐treated sample)/FI of glycated sample] × 100.

#### 2.2.6. Investigation of AGEs-Specific Amide-I Band via FTIR Spectral Studies

The FTIR analysis of native and Gly-BSA with and without (5, 25 and 50 *μ*M) GA and AG was documented on Perkin Elmer Spectrum version 10.03.06 [4].

#### 2.2.7. CD Spectral Analysis

Far-UV circular dichroism (CD) analysis of all the samples at varying concentrations with and without inhibitors was executed with a J-815 Jasco spectropolarimeter [4, 5]. The results were read by K2D2 software (http://cbdm-01.zdv.uni-mainz.de/~andrade/k2d2/).

#### 2.2.8. TNBS Assay to Determine Lysine Modification

Lysine content in glycation modified and unmodified albumin in the presence or absence of GA (5, 25, and 50 *μ*M) and AG (50 *μ*M) were determined by using TNBS as described earlier. The absorbance was recorded at 346 nm using blank [5, 28]. Percentage of free lysine residue was estimated via the following calculation: %Free lysine residues = [(Abs.of Gly‐BSA–Abs.of Native BSA or GA‐/AG‐treated Gly‐BSA)/Abs.of Gly − BSA] × 100.

#### 2.2.9. Analysis of Free Arginine Residues

Quantification of arginine modification in unglycated, glycated, and GA-/AG-treated glycated samples was determined via phenanthroquinone as mentioned previously [5, 29]. The FI of the above samples was evaluated by exciting the samples at 312 nm, and the emission spectra were measured from 350-450 nm.

#### 2.2.10. Detection of Protein Aggregation via Congo Red Binding Assay

The protein aggregation in unmodified BSA, glycated, and GA-/AG-treated glycated BSA samples was measured using a specific dye for amyloid detection, i.e., CR. The CR binding assay was determined by the method previously described [5, 30]. Briefly, 100 *μ*L (pH 7.4) CR solutions (100 *μ*M) was mixed with 500 *μ*L of the abovementioned samples and incubated for 20 min. The absorbance was recorded from 400 to 700 nm (wavelength) for all the samples.

#### 2.2.11. Measurement of Amyloid Fibrils Using ThT Fluorescence Assay

The protein fibrillation was calculated using ThT, a benzothiazole dye. This dye exhibits increased fluorescence when interacting with amyloid fibrils. Therefore, the amyloid fibril formation was evaluated in the current study in all the samples mentioned above using a ThT method as described previously [31]. The Agilent Cary Eclipse Spectrofluorimeter was used to measure the FI. The FI was calculated by exciting the samples at 440 nm, and the emission spectra were measured from 460 to 600 nm [5]. The percentage variation in FI for each sample was quantified as %Increase/Decrease in FI = [(FI of glycated sample − FI of native or GA‐/AG‐treated sample)/FI of glycated sample] × 100.

#### 2.2.12. ANS Fluorescence Spectral Analysis

The ANS is a well-known extrinsic fluorescent dye that emits significantly more fluorescence due to the hydrophobicity of a binding position and the constrained mobility of ANS, as a result of structural changes and when attaching to protein clumps [5, 32]. ANS binding with all the samples mentioned above was evaluated by using the previously published method [33]. ANS concentration used was 5 *μ*M. ANS fluorescence was measured at 525 nm via following equation, and the results have been denoted as arbitrary units (a.u.): %Increase/Decrease in ANS FI = [(FI of Gly‐BSA − FI f unmodified or inhibitor‐treated Gly‐BSA)/FI of Gly‐BSA] × 100.

#### 2.2.13. Molecular Docking Studies of D-Ribose, GA and AG with BSA

The PDB structure of the BSA (PDB ID: 4F5S) was extracted from Brookhaven Protein Data Bank, http://www.rcsb.org, and energy minimized, in order to conduct molecular docking tests. The ligands D-ribose (PubChem ID: 10975657), GA (PubChem ID: 14982) and AG (PubChem ID: 2146) were exported separately from the PubChem database, https://pubchem.ncbi.nlm.nih.gov/, as particular sdf files for docking experiments against BSA. *In silico* docking was executed by PyRx Autodock vina as described in previous reports [34–36].

#### 2.2.14. Statistical Analysis

For entire biochemical measurements, samples were taken in triplicate, and statistics was provided as mean ± SEM. ANOVA was used to test statistical significance via GraphPad Prism version 4.02 for Windows (GraphPad App, San Diego, USA) as mentioned in earlier reports [8, 37].

## 3. Results

### 3.1. Effect of GA on In Vitro BSA-AGE Formation

The data illustrated in Figure 1(c) clearly depicted that D-ribose (80 mM) caused 85.79% BSA glycation that results in augmented hyperchromicity (18^th^ day). However, addition of Gly-BSA with 5, 25, and 50 *μ*M GA illustrated a marked decrease in hyperchromicity with a maximum decline of 12.35% detected in 50 *μ*M-treated sample, when matched to the Gly-BSA. AG (standard drug), at 5, 25 and 50 *μ*M concentration, also displayed declined hyperchromicity with 45.17%, 24.2%, and 17.24%, in Gly-BSA-administered samples, respectively, when compared to the Gly-BSA sample (Figure 1(c)).

### 3.2. GA Reduces the Level of EGPs in Gly-BSA

**Figure 2:**
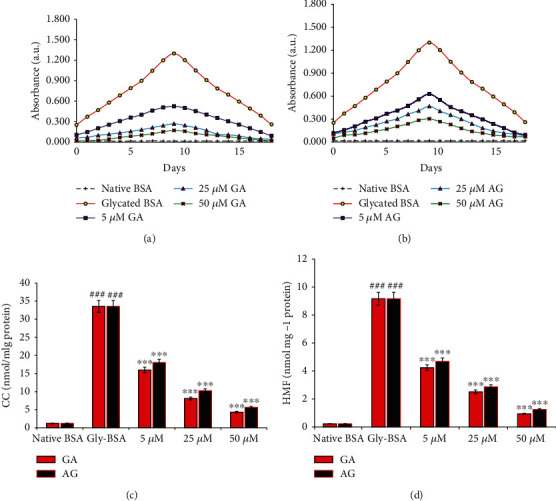
(a, b) Effect of GA and AG on NBT reduction. The data is representative of the mean ± SEM of three independent assays. (c) Carbonyl content in native BSA, Gly-BSA, and GA-/AG-treated Gly-BSA. The data represented the mean ± SEM of three independent experiments. Significant difference vs. native BSA at ^###^*p* < 0.001. Significant difference vs. Gly-BSA at ^∗∗∗^*p* < 0.001. (d) HMF content of native BSA, Gly-BSA, and GA-/AG-treated Gly-BSA. The data represented the mean ± SEM of three independent experiments. Significant difference vs. native BSA at ^###^*p* < 0.001. Significant difference vs. Gly-BSA at ^∗∗∗^*p* < 0.001.

NBT reduction assay was used to measure the ketoamines/EGPs/Amadori product colorimetrically. During incubation (9^th^ day), BSA alone showed insignificant ketoamine, while Gly-BSA had a maximum ketoamine level (98.46%). After 9 days of incubation, the ketoamines/Amadori products began transforming into AGEs. However, treatment of Gly-BSA with 5, 25, and 50 *μ*M GA significantly decreased the ketoamine content by 59.53%, 79.15%, and 90.30%, respectively. On the contrary, in AG-treated Gly-BSA, the ketoamine level was also decreased but to a lesser extent than GA, which is only 6.61% at 50 *μ*M (Figures 2(a) and 2(b)).

### 3.3. GA Scavenges Protein-Bound Carbonyl Contents

Nonenzymatic glycation leads to elevated protein-bound carbonyl level, a remarkable protein oxidation biomarker [1, 4]. From outcomes presented in Figure 2(c), it was evident that BSA glycated with D-ribose displayed an obvious rise in CC by 96.39% compared to the native BSA which was prominently diminished in the presence of GA in a dose-dependent manner. The carbonyl scavenging potential of GA was observed to be 87.11% at 50 *μ*M in GA-treated Gly-BSA samples. Moreover, AG also reduced the bound carbonyl groups by 46.24%, 69.48%, and 83.20% in 5, 25, and 50 *μ*M-treated samples, respectively.

### 3.4. GA Declines the HMF Content in Gly-BSA

HMF content level was estimated to be highest on 9^th^ day by 99.76% in Gly-BSA, whereas the low HMF content level was observed in native BSA. On the other hand, addition of GA (5, 25, and 50 *μ*M) considerably reduced the HMF content level by 53.87%, 72.62%, and 89.85%, respectively. Treatment with AG also showed decreased HMF content by 48.85%, 68.81%, and 86.47% in 5, 25, and 50 *μ*M-treated Gly-BSA samples, respectively, when matched with Gly-BSA (Figure 2(d)).

### 3.5. GA Reduces the Formation of Fluorescent BSA-AGEs

**Figure 3:**
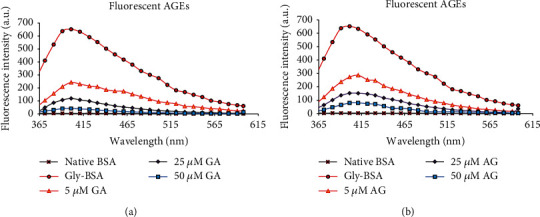
(a) Fluorescence emission spectra of native BSA, Gly-BSA, and GA-treated Gly-BSA samples. All the samples were excited at 370 nm, and the emission spectra were recorded between 360 and 600 nm on Agilent Cary Eclipse spectrofluorimeter at 25 ± 0.1°C. The data represented the *mean* ± *SEM* of three independent experiments. (b) Fluorescence emission spectra of native BSA, Gly-BSA, and AG-treated Gly-BSA samples. All the samples were excited at 370 nm, and the emission spectra were recorded between 360 and 600 nm on Agilent Cary Eclipse spectrofluorimeter at 25 ± 0.1°C. The data represented the mean ± SEM of three independent experiments.

In order to assess fluorescent AGE inhibition, fluorescence spectroscopy was done in all samples. The excitation of samples was done at 370 nm, and the emission spectrum was detected in the wavelength array of 350-600 nm. Our results showed that native BSA did not exhibit any emission spectra, while Gly-BSA exhibited a marked rise of 99.41% in fluorescence intensity (FI). However, GA significantly decreased FI with marked inhibition of 93.70% observed at 50 *μ*M, while AG-treated Gly-BSA samples (5, 25, and 50 *μ*M) also exhibited a decline in FI by 64.4%, 79.9%, and 90.23%, respectively, in comparison to Gly-BSA (Figures 3(a) and 3(b)).

### 3.6. GA Restores Amide-I Bond in Gly-BSA

**Figure 4:**
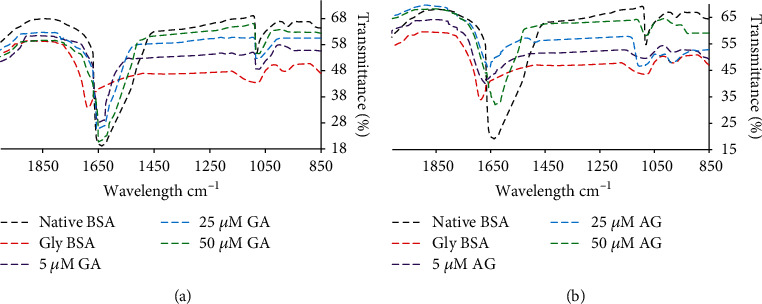
(a) FTIR spectral analysis to determine amide-I band delocalization of native BSA, Gly-BSA, and GA-treated Gly-BSA samples. (b) FTIR spectral analysis to determine amide-I band delocalization of native BSA, Gly-BSA, and AG-treated Gly-BSA samples. The data represented the mean ± SEM of three independent experiments for each sample.

The structural modification in protein, i.e., the position of amide-I band in native BSA and Gly-BSA as well as in the presence or absence of GA/AG was determined by FTIR spectral analysis. The result presented in Figure 4(a) showed that Gly-BSA observed an alteration in peak position of amide-I band from 1636.93 to 1690.52 cm^−1^, while the addition of different concentrations of GA markedly reinstated the peak position of the amide-I band, when matched with glycated BSA. On the other hand, 50 *μ*M AG treatment also restored the alteration in Gly-BSA to1652.14 cm^−1^ (Figure 4(b)).

### 3.7. GA Restores the Structural Alterations in Gly-BSA

**Figure 5:**
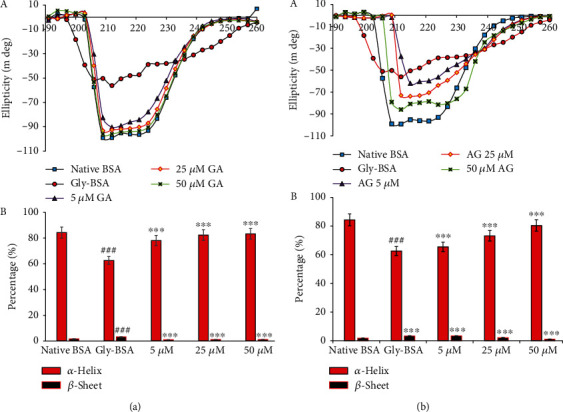
(a, A) GA preserves the secondary structures in D-ribose-mediated protein glycation. Far UV CD (190-260 nm) spectra of native BSA, Gly-BSA, and GA-treated Gly-BSA samples. The spectra are the mean ± SEM of three determinations. The results are represented as change in ellipticity (mdeg) of Gly-BSA either in the presence or absence of GA. (a, B) The % *α*-helix and *β*-sheet content of native BSA, Gly-BSA, and GA-treated Gly-BSA. % *α*-helix and *β*-sheet content was interpreted through K2D2 software (http://cbdm-01.zdv.uni-mainz.de/~andrade/k2d2/). Values (% *α*-helix and *β*-sheet content) are the mean ± SEM of three determinations. Significant difference vs. native BSA at ^###^*p* < 0.001. Significant difference vs. Gly-BSA at ^∗∗∗^*p* < 0.001. (b, A) AG preserves the secondary structures in D-ribose-mediated protein glycation. Far UV CD (190-260 nm) spectra of native BSA, Gly-BSA, and AG-treated Gly-BSA samples. The spectra are the mean ± SEM of three determinations. The results are represented as change in ellipticity (mdeg) of Gly-BSA either in the presence or absence of AG. (b, B) The % *α*-helix and *β*-sheet content of native BSA, Gly-BSA, and AG-treated Gly-BSA. Values (% *α*-helix and *β*-sheet content) are the mean ± SEM of three determinations. Significant difference vs. native BSA at ^###^*p* < 0.001. Significant difference vs. Gly-BSA at ^∗∗∗^*p* < 0.001.

Further, secondary structural changes were also measured via CD spectral studies, which depicted that secondary structure, i.e., *α*-helix (84.3 ± 1.79%) and *β*-sheet (1.72 ± 0.17%) of native BSA remained unchanged, whereas Gly-BSA showed modification in both *α*-helix i.e., from 84.3 ± 1.79% to 62.6 ± 1.63%) and *β*-sheet, i.e., from 1.78 ± 0.14% to 3.79 ± 0.25%). Though, when treated with different doses (5, 25, and 50 *μ*M) of GA significantly restored the structural alteration of *α*-helix and *β*-sheet to a normal level in a concentration-dependent manner. The restoration in structural alteration was observed to be maximum in 50 *μ*M-treated samples in both *α*-helix (83.53 ± 1.75%) and *β*-sheet (1.74 ± 0.11%) (Figure 5(a)). Moreover, 50 *μ*M AG also restored both *α*-helix (from 62.6 ± 1.63 to 69 ± 1.71%) and *β*-sheet (from 3.79 ± 0.21 to 2.89 ± 0.16%), but to a lesser extent than GA when compared to Gly-BSA (Figure 5(b)).

### 3.8. Effect of GA on Modification of Free Lysine Residues

**Figure 6:**
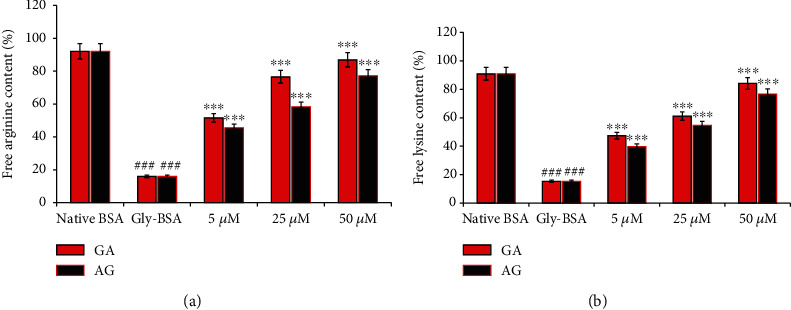
(a) Free arginine content of native BSA, Gly-BSA, and GA-/AG-treated Gly-BSA samples. The data shown in graph is the mean ± SEM of three independent experiments. Significant difference vs. native BSA at ^###^*p* < 0.001. Significant difference vs. Gly-BSA at ^∗∗∗^*p* < 0.001. (b) Free lysine content of native BSA, Gly-BSA, and GA-/AG-treated samples. The data represented the mean ± SEM of three independent experiments. Significant difference vs. native BSA at ^###^*p* < 0.001. Significant difference vs. Gly-BSA at ^∗∗∗^*p* < 0.001.

Lysine residues in native BSA and Gly-BSA with and without GA and AG were estimated by TNBS assay.  Figure 6(a) showed that Gly-BSA exhibited a significant decrease of 15.34% free lysine content in comparison with the corresponding BSA value. This decrease in the percentage of free lysine content was markedly increased by varying concentrations (5, 25, and 50 *μ*M) of GA-treated Gly-BSA. The maximum increase in percentage lysine content was found to be in 50 *μ*M by 84.08%, followed by 25 *μ*M (61.12%) and 5 *μ*M (47.4%), respectively. Moreover, AG also showed an increase in percentage lysine content by 39.72%, 54.63%, and 76.53% in 5 *μ*M-, 25 *μ*M-, and 50 *μ*M-treated Gly-BSA samples, respectively.

### 3.9. Effect of GA on Free Arginine Residues in Gly-BSA

The native BSA and Gly-BSA with and without GA and AG were also assayed for free arginine residues as determined by phenanthrenequinone assay. The level of arginine was 92.12% in native BSA and 16.03% in Gly-BSA. However, treatment with 5, 25, and 50 *μ*M of GA simultaneously augmented free arginine level with major increase in the percentage of reacted arginine residues was observed in 50 *μ*M (86.99%) followed by 25 *μ*M (76.63%) and 5 *μ*M (51.59%). The standard drug AG at 5, 25, and 50 *μ*M concentrations also increase the arginine content by 42.5%, 58.3%, and 77.1%, respectively (Figure 6(b)).

### 3.10. GA Restrain Glycation Induced Aggregation and Fibrillation of BSA

**Figure 7:**
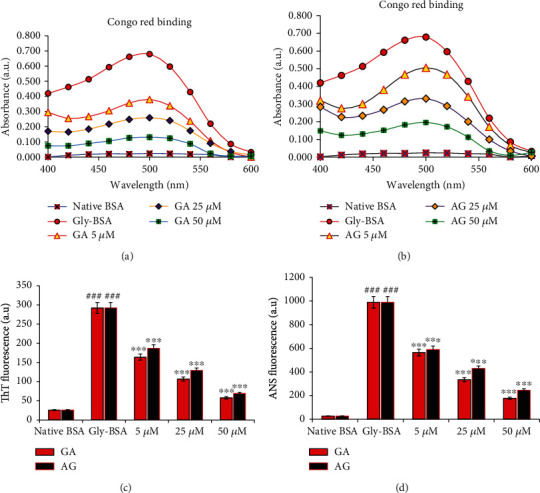
(a, b) Congo red binding assay of native BSA, Gly-BSA, and GA- and AG-treated Gly-BSA. Absorbance was recorded in the range of 400-700 nm, and the data is the average of three determinants. The data represented the mean ± SEM of three independent experiments. (c) Thioflavin T- (ThT-) specific extrinsic fluorescence of native BSA, Gly-BSA, and GA-/AG-treated Gly-BSA samples. The samples were excited at 370 nm, and the emission was recorded at 485 nm. The data represented the mean ± SEM of three independent experiments. Significant difference vs. native BSA at ^###^*p* < 0.001. Significant difference vs. Gly-BSA at ^∗∗∗^*p* < 0.001. (d) ANS-specific fluorescence spectra of native BSA and Gly-BSA in the presence or absence of varying concentrations of GA and AG. The samples were excited at 380 nm, and the emission was recorded between 500 and 600 nm. The data represented the mean ± SEM of three independent experiments. Significant difference vs. native BSA at ^###^*p* < 0.001. Significant difference vs. Gly-BSA at ^∗∗∗^*p* < 0.001.

The extent of formation of aggregates during glycation was determined by CR binding assay. In this background, we observed that Gly-BSA depicted an 96.46% increase in CR-specific absorption compared to the native BSA. Addition of different concentrations (5, 25, and 50 *μ*M) of GA significantly decreased the CR-specific absorbance with major decline of 80.70% was observed after addition of 50 *μ*M GA to Gly-BSA samples followed by 25 *μ*M (61.85%) and 5 *μ*M (44.03%)-treated Gly-BSA samples, respectively. Moreover, AG also exhibited the similar pattern but the reduction was less (71.13%) as compared to GA-treated Gly-BSA at 50 *μ*M concentration (Figures 7(a) and 7(b)). Our result here also depicted that Gly-BSA showed a significant rise of 91.09% in ThT-specific fluorescence when matched with the corresponding native BSA. This significant upsurge in ThT-specific fluorescence was markedly decreased in GA-administered Gly-BSA samples. The maximum reduction in Tht-specific fluorescence was observed in 50 *μ*M (80.13%) of GA-treated Gly-BSA samples. Also, reference drug whereas AG illustrated a reduction 76.36% at same concentration, compared to Gly-BSA (Figure 7(c)).

### 3.11. Impact of GA on ANS Binding in Gly-BSA

Figure 7(d) showed that Gly-BSA resulted in a major increase in ANS particular fluorescence by 97.37%, when matched with the native BSA. However, we reported that treatment with GA resulted in marked decrease in ANS FI with highest decrease of 82% observed in sample treated with 50 *μ*M GA. Moreover, AG too illustrated a decreased in the ANS fluorescence by 40.34%, 56.62%, and 75.22% in Gly-BSA samples treated with 5, 25, and 50 *μ*M, respectively, compared to the corresponding native BSA.

### 3.12. In Silico Study on Molecular Interaction of GA with BSA

**Figure 8:**
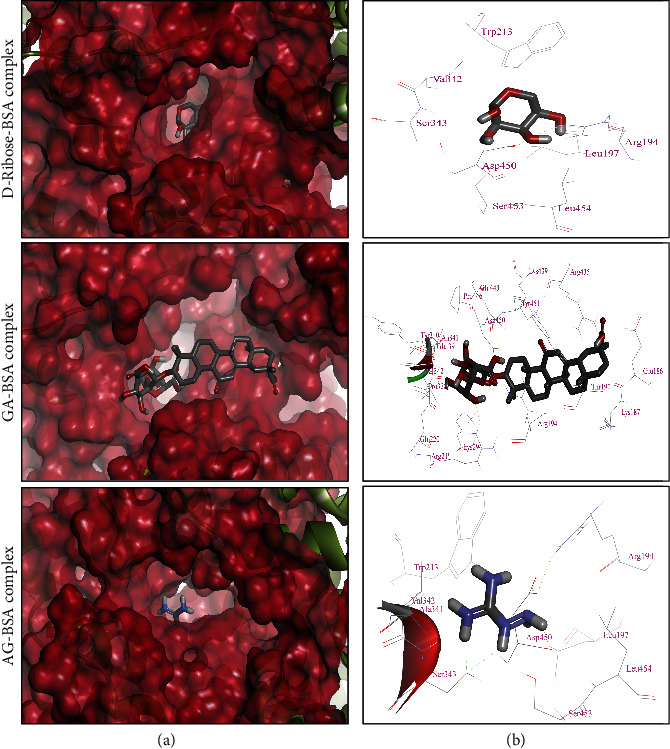
Molecular binding of D-ribose, GA, and AG with BSA (PDB ID: 4F5S). (a) Binding of GA (*Δ*G: -8.8 Kcal/mol), AG (*Δ*G: -4.3 Kcal/mol), and D-ribose (*Δ*G: -5.2 Kcal/mol) in the binding pocket of BSA while BSA has been represented as hydrophobic surface. (b) Enlarged view of interaction of D-ribose, GA, and AG with the interacting residues of BSA.

In order to validate the *in vitro* antiglycation potential of GA, we used computer-aided molecular docking approach and reported that GA showed strong binding with BSA (binding energy, *Δ*G, of -8.8 Kcal/mol), whereas, the binding of AG to the BSA was comparatively weaker (*Δ*G: -4.3 Kcal/mol), when matched with that of GA. The binding of GA with BSA involved its interaction with the Glu186, Lys187, Thr190, Arg194, Arg217, Gln220, Lys294, Pro338, Glu339, Tyr340, Ala341, Val342, Arg435, Lyr436, Pro446, Asp450, and Tyr451, whereas AG-BSA complex was stabilized by the interaction with Arg194, Leu197, Trp213, Ala341, Val342, Ser343, Asp450, Ser453, and Leu454. Moreover, D-ribose also interacted with identical residues as reported in AG-BSA-complex formation (*Δ*G: -5.2 Kcal/mol). Most importantly, both GA and AG interacted with BSA at similar sites and their interaction shared common residues Arg194, Ala341, Val342, and Asp450; hence, participation of common residues during binding of GA and D-ribose with BSA signifies the competitive inhibition of glycation process (Figure 8).

## 4. Discussion

The posttranslational alteration of proteins or amino acids through reducing sugars such as D-ribose is termed as nonenzymatic glycation that results in generating EGPs and irreversible heterogenous by-products known as AGEs [1]. AGE formation, accumulation, and interaction with their receptors, i.e., RAGE are considered to demonstrate a noteworthy part in diabetes-related secondary complications as well as in development of Alzheimer's disease and cardiovascular disease (CVD) [2, 8, 38, 39]. Several molecular strategies have been proposed to counteract AGE formation and accumulation, particularly, based on inhibiting the development of EGPs and AGEs, carbonyl scavenging, AGEs-RAGE signaling, or interference with cellular effects of AGEs [1, 5, 8, 40]. Proteins present in serum are frequently observed to undergo nonenzymatic glycation. Albumin, the most abundant human plasma protein, is a common target which contains three domains (I, II, and III), and among these, domains II and III are known to be major binding regions for drugs like warfarin and ibuprofen [11]. Any alteration in the albumin structure can disturb its drug/molecule binding affinity which in turn has profound consequences on therapeutic modulations in diabetic patients. Glycation may also affect the persistence as well as plasma protein binding efficacy of different drugs, thus making the inhibition of glycation-induced modification of macromolecules a major therapeutic strategy in metabolic syndromes [2, 41].

Several small molecules from natural sources (i.e., iridin and tocotrienol) or chemically synthesized compounds that inhibit any one of the above glycation pathways are now gaining significant attention owing to their beneficial ability to minimize incidence and death associated with diabetes-related complications and other pathogenesis [4, 5, 42]. This is the preliminary study that reports the inhibitory activity of GA against the formation of EGPs and AGEs and the protective effect towards glycation-induced multiple protein modifications which was based on various physicochemical and in silico molecular interaction studies.

The results from the current study clearly depicted that D-ribose-induced BSA glycation led to a substantial rise in hyperchromicity due to the generation of glycation adducts (particularly AGEs) and glycation-induced alteration of the protein conformers, which was markedly supressed by GA treatment. This protective effect of GA might be due to GA's interference with the early attachment of carbonyl group of D-ribose with the free amino groups of protein, hence inhibiting Gly-BSA formation [5, 11]. During the intermediate stage of glycation, unsteady, reversible Schiff bases are produced that contribute in the development of EGPs, which is clinically used as an indicator for glycemic indexing in the identification of diabetic subjects [5]. The protective effects of GA on D-ribose-induced protein modifications are well justified by previous reports demonstrating the beneficial effects of other natural compounds, i.e., iridin and tocotrienol [4, 5].

Ketoamine estimation is a widely adopted method that measures level of EGPs/Amadori products. Thus, the NBT method was used for assessing the protective effect of GA against EGPs [43, 44]. From our results, we observed that the level of EGPs/Amadori product was significantly increased in Gly-BSA; however, addition of varying concentrations of GA reduced the formation of EGPs or Amadori products in a dose-dependent manner. This apparent decline in the absorption can be attributed to the potent antioxidant effects of GA [5, 45, 46] and its ability to mask the amino acid residues that are the more prone to glycoxidative modifications. Therefore, EGP inhibition might be beneficial in evading the highly reactive and lethal AGEs [44].

Furthermore, CC, which is a major protein oxidation biomarker, was assessed during the process of glycation. CC is generated during the EGPs or Amadori product formation and contributes to the AGE accretion in tissues [4, 33]. The scavenging of carbonyl compounds by natural and synthetic agents has been shown to be potent method for inhibition of nonenzymatic glycation reaction [4, 5]. In the current work, protein CC and HMF level was increased in the Gly-BSA sample, and upon treatment with GA, it was evident that the CC and HMF level was considerably reduced. Hence, the above explanations advocate the beneficial function of GA as a powerful carbonyl scavenger or against these EGPs, which might be due to its free radical scavenging activity [44].

It is well known that fluorescence and cross-linking are some of the characteristics of AGEs [4, 8]. Therefore, autofluorescence was used to assess the formation of fluorescent AGEs when Amadori products or EGPs were found to be diminished, an indication for the transition of EGPs into AGEs [38]. Our results reported that Gly-BSA showed an increased FI and the level of fluorescent-AGEs was greatly reduced in a dose-dependent manner in GA-treated Gly-BSA samples, which might be due to GA's antioxidant potential resulting in the diminution of fluorogenic AGEs, thus, increasing the protein stability [44]. These findings are well in accordance with the previous reports depicting the inhibitory effects of natural compounds against fluorogenic AGE formation [4, 5].

Furthermore, glycation also causes conformational changes in the proteins resulting in a secondary structural change from helical to beta-sheet conformers. In this context, Far UV-CD spectrum study was performed to evaluate the structural changes in Gly-BSA before and after treatment with GA. Our results depicted the structural shift and changeover in both *α*-helix and *β*-sheets in Gly-BSA, resulting in substantial damage and instability in protein secondary structure, which is similar to the results documented in earlier published reports [4, 5]. However, it was also observed that treatment with GA markedly protected both the *α*-helix and *β*-sheets against glycation-induced conformational alterations in albumin which might be due to the diminished level of AGEs as well as reactive oxygen species (ROS) in GA-treated BSA samples. In contrast, AG also protected structural changes but to a lesser extent than GA which may be due to its prooxidant nature [5, 47]. Hence, these results inferred strong *in vitro* antiglycating ability for GA because GA could have interacted with BSA and inhibited dicarbonyl and ROS formation during protein oxidation and loss of secondary structure of the protein [4, 5].

FTIR is also one of the sophisticated methods opted for analyzing the protein secondary structural changes, and the FTIR spectra contain amide-I, C = O and amide-II, N-H bonds in the range of 1500–1700 cm^−1^ [48, 49]. In the same context, our FTIR spectral studies also demonstrated the shift in amide-I band position as well as increased transmission intensities in D-ribose-induced Gly-BSA, when matched to unmodified BSA. Such structural perturbations due to the delocalization of amide-I band of Gly-BSA were markedly restored by GA treatment. These findings are well in agreement with previously published reports [4, 5]. In comparison, 50 *μ*M AG exhibited somewhat lesser improvement in amide-I peak position in Gly-BSA that may be owing to AG's prooxidant behaviour against proteins at low concentrations [5, 50].

Proteins having higher Lys and Arg contents are considered to be more vulnerable to glycation modification leading to the progression of some AGEs such as carboxyethyl lysine (CEL), carboxymethyl lysine (CML), and vesperlysine (VESP) [1]. Mass spectral analysis has revealed that BSA contains 583 residues and 59 of which are Lys and 23 are Arg and that serve as prone glycation sites [5, 44]. Thus, estimating the level of free lysine and arginine might provide information about the protective effects of GA against glycation. In the current study, free Lys and Arg levels were significantly decreased in case of Gly-BSA which depicts their engagement and covalent modifications with D-ribose leading to the formation of EGPs and AGEs [5]. However, simultaneous treatment of Gly-BSA with GA markedly alleviated the free Lys and Arg residues and this reduced interaction between D-ribose and Lys/Arg residues could have possibly resulted in restricted glycation, ROS generation, and di-carbonyl-mediated protein oxidation [5, 51, 52].

Nonenzymatic glycation is well known to modulate protein structure and induces aggregation and fibrillation [5, 52] which is an important phase in the prognosis and growth of various neurological diseases as well as diabetic complications [1, 52]. Most commonly, the CR dye is used for the detection/characterization of amyloid aggregates, whereas ThT dye is commonly implied to characterize amyloid fibrils [53]. The data from our study depicted that Gly-BSA exhibited higher binding affinity for CR as well as the ThT-specific fluorescence was also higher in Gly-BSA, when compared to unmodified BSA. These findings validated the hypothesis that glycation leads to the development of amyloids and fibrillar accretion which could also be correlated with the pathogenesis of neurological and diabetic complications. Our findings are in well agreement with the earlier available reports supporting the use of CR and ThT dyes for the characterization of protein aggregation [54–56]. Nevertheless, we observed that the presence of GA in the glycation milieu markedly protected against the formation of amyloids and fibrillar accretion in BSA as evident by diminished CR binding as well as ThT-specific fluorescence assays. Recently, iridin, a 7-glucoside of irigenin and a natural antioxidant, has also been reported with similar protective activities [5].

It is apparent from the above results that GA can inhibit the protein damage and aggregation caused by AGEs most specifically by interacting with glycation susceptible positions of proteins, i.e., lysine and arginine. Similarly, various dyes, such as ANS, exhibiting extrinsic fluorescence phenomenon, are commonly known to emit fluorescence once exposed to the hydrophobic areas, particularly inner apolar surfaces of the proteins once their counterparts are exposed following the protein modifications [5, 47]. In continuation, we also reported that Gly-BSA possesses higher ANS-specific fluorescence which confirms the structural transition and unfolding patterns in Gly-BSA that eventually exposed the Gly-BSA's interior apolar surfaces and allowed ANS to combine its hydrophobic milieu. However, such structural alterations and protein unfolding were markedly decreased in GA-treated Gly-BSA with the maximum reduction observed in Gly-BSA treated with 50 *μ*M GA. These results are consistent with the previously published reports demonstrating the protective effects of natural compounds against di-carbonyl-induced protein modifications [5, 52]. This overall protective effect of GA against glycation-mediated protein modifications could be attributed to its antioxidant and anti-AGEs capability, allowing GA to scavenge the free radicals and consequently attenuate glycation-induced multiple protein modifications.

In addition to abovementioned *in vitro* physiochemical experiments, the molecular interaction studies of GA and AG were also performed to explore the binding patterns of these compounds to form GA-BSA and AG-BSA complexes, respectively. The results from our molecular docking analysis showed that GA forms a more stable complex with BSA than AG, as evident by their binding energies (*Δ*G: -8.8 Kcal/mol and *Δ*G: -4.3 Kcal/mol, respectively). It is well reckoned that presence of Lys and/or Arg residues makes proteins more susceptible to glycation-mediated oxidative modifications and consequently leads to structural changes and altered functionality [1, 5]. Therefore, masking these Lys and Arg residues by different synthetic or natural inhibitors may become a key mechanism for antiglycation therapeutics. Similarly, the most significant result of our in silico research was that the binding of GA with BSA involved three Lys residues (Lys187, Lys294, and Lys436) and three Arg residues (Arg194, Arg217, and Arg435), which are the most prone sites for the glycation of BSA. In contrast, no Lys residue was involved in the binding of AG with BSA, while it involved only one Arg residue of BSA which is also considered the preferred site for glycation (Arg194). These findings revealed that GA is more a potent antiglycation agent than AG as it binds to BSA with higher binding energy than AG as well as one molecule of GA occupies more Lys and Arg residues of BSA and challenges D-ribose for available sites on BSA for glycation.

In contrast, binding of AG with BSA was weaker than GA and it masks only one preferred glycation site of the BSA (Arg194). These findings suggest that the antiglycation potential of GA might be attributed to its antioxidant potential [45] as well as its ability to mask higher number of preferred glycation sites of the BSA than AG. Our results from *in silico* molecular modelling studies are consistent with the previous report that demonstrated that eugenol exhibits high binding affinity for surface Lys residues on albumin resulting in protection against glycation-induced protein modifications [12]. The binding pattern of GA against BSA in the current study is well supported by the high affinity interaction of GA as well as other terpenoids, i.e., lycopene preferably with the hydrophobic residues of distinct molecular targets, i.e., high mobility group box 1 [57], HMG-CoA reductase [34], and SARS-CoV-2-specific spike glycoprotein and Nsp-15 [58].

## 5. Conclusion

In summary, the above results are initial demonstration of GA-mediated fortification against *in vitro* D-ribose-induced nonenzymatic glycation of albumin. Being a free radical scavenger, GA exerted protection at multiple stages of glycation reaction via inhibiting the generation of EGPs and AGEs, fluorescent AGEs, scavenging protein CC and HMF content, and protection against D-ribose-induced modification of BSA as evident by increased free Arg and Lys residues in GA-treated Gly-BSA samples. Moreover, GA also attenuated D-ribose-induced alterations in the secondary structure of BSA by protecting the *α*-helix and *β*-sheets and amide-I band delocalization. In addition, GA also inhibited the protein damage and aggregation caused by AGEs as evident by reduced CR binding and ANS as well as ThT-specific fluorescence, which might be achieved via interaction of GA with glycation-susceptible sites of BSA. Moreover, our *in vitr*o findings are well justified by in silico data which further revealed that masking of Lys and Arg residues by GA is perhaps another insight accountable for effective antiglycation property of GA. Based on this, we concluded that GA possesses significant antiglycation activity and future *in vivo* and large-scale clinical studies are needed to establish its role as an antiglycation therapeutic agent.

## Data Availability

The data used to support the results of the study are contained in this article.
